# Life Cycle Dominates the Volatilome Character of Dimorphic Fungus *Coccidioides* spp.

**DOI:** 10.1128/mSphere.00040-21

**Published:** 2021-04-14

**Authors:** Emily A. Higgins Keppler, Heather L. Mead, Bridget M. Barker, Heather D. Bean

**Affiliations:** a School of Life Sciences, Arizona State University, Tempe, Arizona, USA; b Center for Fundamental and Applied Microbiomics, The Biodesign Institute, Arizona State University, Tempe, Arizona, USA; c Pathogen and Microbiome Institute, Northern Arizona University, Flagstaff, Arizona, USA; University of Georgia

**Keywords:** GC×GC, Valley fever, biomarkers, coccidioidomycosis, comprehensive two-dimensional gas chromatography, dimorphic fungus, fungal infections, untargeted metabolomics, volatile metabolites

## Abstract

Valley fever (coccidioidomycosis) is an endemic fungal pneumonia of the North and South American deserts. The causative agents of Valley fever are the dimorphic fungi Coccidioides immitis and C. posadasii, which grow as mycelia in the environment and as spherules within the lungs of vulnerable hosts. Current diagnostics for Valley fever are severely lacking due to poor sensitivity and invasiveness, contributing to a 23-day median time to diagnosis, and therefore, new diagnostic tools are needed. We are working toward the development of a breath-based diagnostic for coccidioidomycosis, and in this initial study, we characterized the volatile metabolomes (or volatilomes) of *in vitro* cultures of *Coccidioides*. Using solid-phase microextraction (SPME) and comprehensive two-dimensional gas chromatography coupled to time of flight mass spectrometry (GC×GC-TOFMS), we characterized the volatile organic compounds (VOCs) produced by six strains of each species during mycelial or spherule growth. We detected a total of 353 VOCs that were at least 2-fold more abundant in a *Coccidioides* culture than in medium controls and found that the volatile metabolome of *Coccidioides* is more dependent on the growth phase (spherules versus mycelia) than on the species. The volatile profiles of C. immitis and *C. posadasii* have strong similarities, indicating that a single suite of Valley fever breath biomarkers can be developed to detect both species.

**IMPORTANCE** Coccidioidomycosis, or Valley fever, causes up to 30% of community-acquired pneumonias in highly populated areas of the U.S. desert southwest where the disease is endemic. The infection is difficult to diagnose by standard serological and histopathological methods, which delays appropriate treatment. Therefore, we are working toward the development of breath-based diagnostics for Valley fever. In this study, we characterized the volatile metabolomes (or volatilomes) of six strains each of *Coccidioides immitis* and *C. posadasii*, the dimorphic fungal species that cause Valley fever. By analyzing the volatilomes during the two modes of growth of the fungus—mycelia and spherules—we observed that the life cycle plays a significant role in the volatiles produced by *Coccidioides*. In contrast, we observed no significant differences in the *C. immitis* versus *C. posadasii* volatilomes. These data suggest that life cycle, rather than species, should guide the selection of putative biomarkers for a Valley fever breath test.

## INTRODUCTION

Coccidioidomycosis, or Valley fever, is a disease that is endemic to the deserts of the western United States, Mexico, and Central and South America and is responsible for an estimated 350,000 new infections per year (1). In highly populated areas of the United States where the disease is endemic, e.g., Phoenix and Tucson, AZ, and the San Joaquin Valley in California, up to 30% of community-acquired pneumonias may be caused by Valley fever, and with growing populations in these regions, Valley fever cases in the United States are expected to climb (2). The causative agent of Valley fever is the opportunistic pathogen *Coccidioides*, a genus of dimorphic fungi comprised of two species, Coccidioides immitis and C. posadasii. In the environment, *Coccidioides* spp. grow in the soil during rainy periods as saprobic mycelia, which become dormant and form arthroconidia during dry periods. As the soil is disturbed, the arthroconidia can become airborne, and when inhaled, the fungus can initiate a parasitic life cycle as spherules within the lungs of a susceptible host, causing pneumonia. The median duration of human Valley fever pneumonia is 120 days, costing over $93,000 per person in direct and indirect costs (2, 3). Approximately three-quarters of the diagnosed cases of Valley fever require antifungal therapy (4), and 1% of those infected will experience disseminated disease, with one-third of those being fatal (4).

A definitive diagnosis of Valley fever requires a positive fungal culture or histological examination of tissue or bodily fluid. However, due to the invasiveness of obtaining suitable lung specimens for culture or histology, serological testing for antibodies against *Coccidioides* is the most commonly performed diagnostic test. Multiple serological tests are available for IgM, IgG, or complement-fixing antibodies, as is an enzyme-linked immunoassay that uses proprietary coccidioidal antigens. However, these tests lack sensitivity for coccidioidomycosis (5, 6). Due to the difficulty in diagnosing Valley fever, it is often mistaken for bacterial pneumonia and inappropriately treated with antibiotics (7–10), and the lack of a suitable diagnostic strongly contributes to an unacceptably long 23-day median time to diagnosis (2).

Breath-based diagnostics are a promising novel approach for diagnosing respiratory infections. Numerous *in vitro* studies have demonstrated that bacterial and fungal respiratory pathogens have unique volatile metabolome (or volatilome) signatures that can be used to differentiate and identify them to the genus, species, and strain levels (11). Thus far, relatively few studies have specifically focused on identifying breath biomarkers for fungal lung diseases. *In vitro* analyses of the volatiles produced by *Aspergillus* spp. have shown that fungi produce many compounds that are also frequently detected in bacterial cultures, e.g., the alcohols ethanol and propanol; the ketones acetone, 2-nonanone, 2-undecanone, and 2,3-butanedione; the sulfides dimethyl disulfide and dimethyl trisulfide; and the pyrazines 2-methyl pyrazine and 2,5-dimethyl pyrazine (12–16). However, *Aspergillus* also produces a wider variety of monoterpenes, sesquiterpenes, and terpenoids, which are less common in bacterial volatilomes (14, 17). With this knowledge, Koo et al. used a biologically guided approach to identify volatile biomarkers of pulmonary invasive aspergillosis (IA), focusing on terpenes and terpenoids produced *in vitro* as putative biomarkers for a breath test (17). They determined that the Aspergillus fumigatus *in vitro* volatilome contained a distinctive combination of monoterpenes and sesquiterpenes, and in breath, the sesquiterpenes were the most specific for differentiating patients with IA from those with other pulmonary infections (17). This example for A. fumigatus establishes a proof of concept that direct detection of fungal metabolites in breath can be used as a noninvasive, species-specific approach to identify the underlying microbial cause of pneumonia.

We are taking an *in vitro*-guided approach toward developing a Valley fever breath test by first building a catalog of volatile compounds produced by C. immitis and *C. posadasii*. In this study, we have cultured six strains of each species and used solid-phase microextraction (SPME) and comprehensive two-dimensional gas chromatography–time of flight mass spectrometry (GC×GC-TOFMS) to characterize the volatile organic compounds (VOCs) produced during mycelial or spherule growth. Although *Coccidioides* almost exclusively grows as spherules in the host, many *in vitro* studies on the pathogenesis and diagnosis of coccidioidomycosis have been performed using mycelial cultures, as the saprobic life cycle has historically been easier to cultivate and maintain (18). We expected to find significant differences in the VOCs between species and between life cycles but also posited that there would be a subset of VOCs that are common to *Coccidioides* spp. during spherule growth, which will serve as our candidate biomarkers for breath test development.

## RESULTS

### *Coccidioides* isolates in this study.

The *Coccidioides* genus is widely distributed throughout North and South America. The genus is composed of two geographically (19) and genetically (19, 20) distinct species, *C. posadasii* and *C. immitis*, which are further divided into five well-defined populations (21–23). *C. posadasii* has a more extensive biogeographic distribution and is divided into three populations, occurring in Texas/Venezuela, Mexico/South America, and Arizona. The species *C. immitis* is divided into two populations found in central and southern California and was recently found in Washington State, representing a newly discovered third distinct population (24, 25). Representative members of all populations cause disease in humans and other mammals (21–23). Due to the magnitude of the observed genetic variation and geographic distribution between species, we hypothesized that metabolomic variation exists. To capture diversity across the volatilome between and within species, we included a total of 12 isolates from both species, representing four populations, all of which were isolated from humans (Table 1).

**TABLE 1:** *Coccidioides* strains used in this study^*a*^

Species	Strain	Population^*b*^	ATCC catalog no.	NCBI accession no.^*c*^
*C. posadasii*	Silveira	AZ	NR-48944	ABAI00000000.2
	B3221	AZ		NA
	B3222	AZ		NA
	RMSCC2343	TX/MEX/SA		SRR3468064
	RMSCC3506	TX/MEX/SA		SRR3468053
	GT-166	TX/MEX/SA		NA

*C. immitis*	RS	SDMX	NR-48942	AAEC00000000.3
	RMSCC2395	SDMX	NR-48938	NA
	RMSCC3505	SDMX		NA
	RMSCC2006	SJV	NR-48934	NA
	RMSCC2009	SJV		SRR3468015
	RMSCC2010	SJV	NR-48935	NA

a All isolates are from human infections.

b Populations are defined as *C. posadasii* collected from Arizona (AZ); *C. posadasii* collected from Texas/Mexico/South America (TX/MEX/SA); *C. immitis* collected from San Diego, CA, and Mexico (SDMX); and *C. immitis* collected from San Joaquin Valley, CA (SJV), as previously reported (46).

c Published genotype data, if available (22). NA, not applicable.

### Characteristics of the *Coccidioides* volatilome by species and life cycle.

We cultured each *Coccidioides* isolate in biological triplicate using temperatures and oxygen concentrations that induce mycelial or spherule growth but using the same culture medium, facilitating direct comparisons between the VOCs produced during the two life cycles. Using SPME to adsorb VOCs from the headspace of fungal culture filtrates and GC×GC-TOFMS to analyze the volatilomes, we detected a total of 353 chromatographic peaks that were at least 2-fold more abundant in a *Coccidioides* culture (*n* = 72) than in the medium controls (*n* = 6) (see Table S1 in the supplemental material). Of the 353 volatiles detected in *Coccidioides* cultures, 28 were identified at level 1 or 2 and therefore were assigned putative names based on mass spectral and chromatographic data (see “Processing and analysis of chromatographic data” in Materials and Methods, below), which included 13 compounds previously associated with human and environmental fungal pathogens (26) (Table 2). For the unnamed compounds, we assigned chemical classifications to 45 of them, which were assigned a level 3 identification based on a combination of mass spectral and chromatographic characteristics (Table S1).

**TABLE 2:** Named *Coccidioides* volatiles and their previous reports in other fungal taxa

VOC	Compound	Functional class	CAS no.	VOC reported in fungal taxon^*a*^							Reference(s)
				1	2	3	4	5	6	7	
29	Acetonitrile	Nitrogen-containing	75-05-8								
97	Acetic acid	Carboxylic acid/ester	64-19-7	X	X						36, 47
147	Acetoin	Ketone	513-86-0		X						47
159	2-Methyl-1-butanol	Alcohol	137-32-6	X	X		X	X	X	X	39, 41, 48–50
195	Hexanal	Aldehyde	66-25-1	X	X						47, 48, 51
196	4-Methyl-3-penten-2-one	Ketone	141-79-7								
206	*N*,*N*-Dimethyl-formamide	Nitrogen-containing	68-12-2								
239	1-Hexanol	Alcohol	111-27-3		X						48
255	2-Hexen-1-ol	Alcohol	2305-21-7								
270	Cyclohexanone	Ketone	108-94-1	X	X	X			X		49, 52
299	1-Ethyl-4-methyl-benzene	Aromatic hydrocarbon	622-96-8								
320	2,5-Hexanedione	Ketone	110-13-4								
332	2-Pentylfuran	Heteroaromatic	3777-69-3	X			X			X	39, 40, 50, 53–55
354	5-Methyl-3-heptanone	Ketone	541-85-5								
359	β-Pinene	Monoterpene	127-91-3	X						X	14, 17, 49, 51
364	4,6-Dimethyl-2-heptanone	Ketone	19549-80-5								
383	Limonene	Monoterpene	138-86-3	X	X		X		X	X	14, 17, 39, 41, 48, 49, 51, 56–58
386	1-Methyl-3-(1-methylethyl)-benzene	Aromatic hydrocarbon	535-77-3								
411	2-Chloro-phenol	Aromatic alcohol	95-57-8								
428	2,4,6-Trimethlpryidine	Heteroaromatic	108-75-8								
435	3-Undecene	Hydrocarbon	1002-68-2								
456	Benzeneacetaldehyde	Aromatic aldehyde	122-78-1		X				X		48, 56
502	1,3-Diethenyl-benzene	Aromatic	108-57-6								
545	2-Decanone	Ketone	693-54-9							X	39
554	2-(2-Butoxyethoxy)-ethanol	Alcohol	112-34-5								
555	Decanal	Aldehyde	112-31-2	X	X						48, 51
820	2-Butyl-1-octanol	Alcohol	3913-02-8								
955	Hexadecane	Hydrocarbon	544-76-3	X							13

a X indicates that the VOC has been previously detected in the fungal taxa associated with the numbered columns, as follows: 1, *Aspergillus* spp.; 2, *Candida* spp.; 3, Cladosporium cladosporioides; 4, *Fusarium* spp.; 5, *Mucor* spp.; 6, *Penicillium* spp.; 7, other species, including environmental fungal species, *Trichoderma* spp., Stropharia rugosoannulata, and Verticillium longisporum.

10.1128/mSphere.00040-21.3 TABLE S1 The 353 *Coccidioides* VOCs detected in the headspace of *in vitro* spherule and mycelial cultures. Columns are defined in the footnotes, in a separate tab. Download Table S1, XLSX file, 0.2 MB. Copyright © 2021 Higgins Keppler et al. 2021 Higgins Keppler et al. https://creativecommons.org/licenses/by/4.0/ This content is distributed under the terms of the Creative Commons Attribution 4.0 International license.

**FIG 1:**
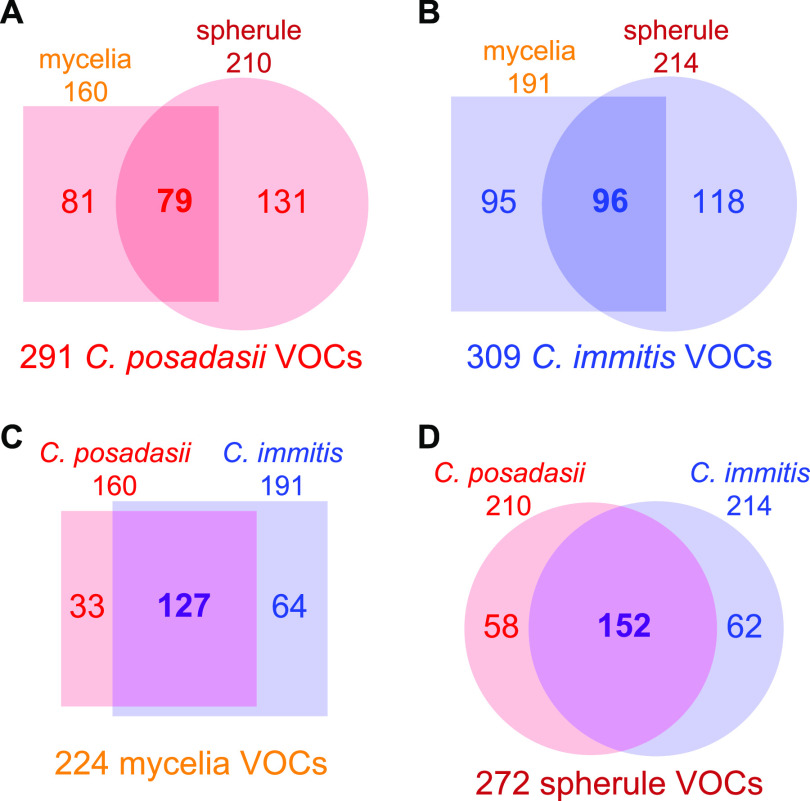
Numbers of analytes detected in at least a 2-fold-higher abundance in a *Coccidioides* culture than in the medium controls that are shared and unique among *C. posadasii* mycelia (square) versus spherules (circle) (A), *C. immitis* mycelia (square) versus spherules (circle) (B), mycelia of *C. posadasii* (red) versus *C. immitis* (blue) (C), and spherules of *C. posadasii* (red) versus *C. immitis* (blue) (D).

The total numbers of volatiles produced by *C. posadasii* and *C. immitis* were similar, at 291 and 309, respectively (Fig. 1; Table S2). Within each species, the two life cycles are quite divergent in their volatilomes, sharing less than one-third of the total volatilome (Fig. 1A and B). However, within each life cycle, the two species share many similarities, with more than one-half of the VOCs being produced by both *C. posadasii* and *C. immitis* (Fig. 1C and D). In aggregate across the genus, or subdivided by species, the spherule volatilome is larger than the mycelial volatilome, but this appears to be driven by considerable differences in volatilome sizes observed in a few isolates (*C. posadasii* B3221 and GT-166 and *C. immitis* RS) (Table S2).

10.1128/mSphere.00040-21.4 TABLE S2 Numbers of volatiles detected in at least a 2-fold-higher concentration in *Coccidioides* cultures than in blank media. The type strains for each species are indicated with an asterisk. Download Table S2, PDF file, 0.1 MB. Copyright © 2021 Higgins Keppler et al. 2021 Higgins Keppler et al. https://creativecommons.org/licenses/by/4.0/ This content is distributed under the terms of the Creative Commons Attribution 4.0 International license.

**FIG 2:**
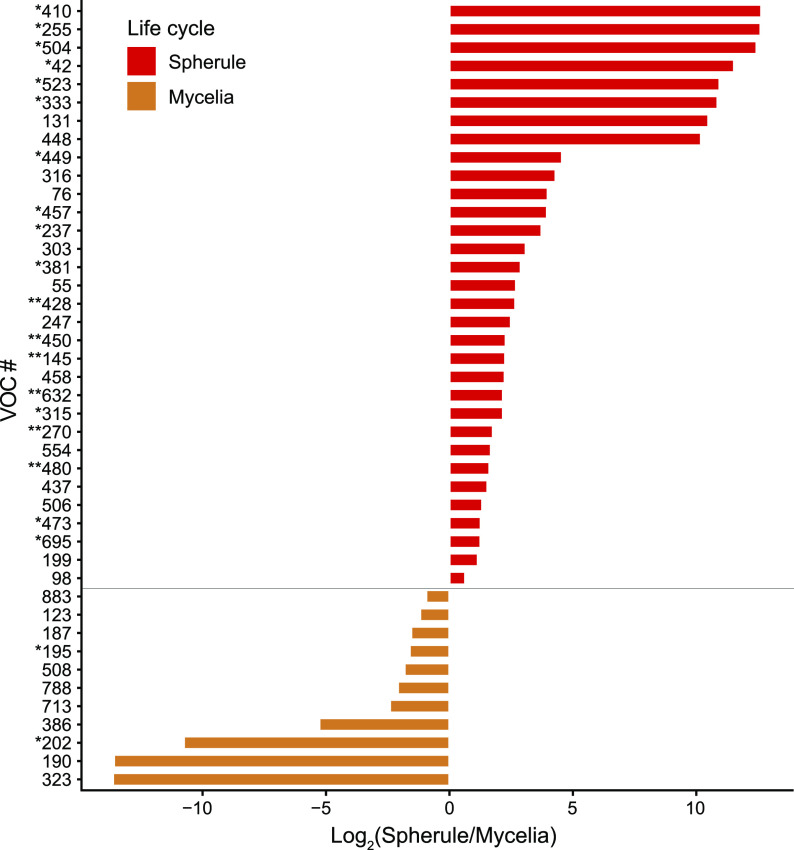
Forty-three volatiles significantly different in abundance between life cycles (*P* < 0.05), expressed as log_2_ fold changes in peak abundance. Volatiles marked with asterisks indicate greater statistically significant differences (*, *P* < 0.001; **, *P* < 0.0001). Compound identifications: 195, hexanal; 255, 2-hexen-1-ol; 270, cyclohexanone; 386, 1-methyl-3-(1-methylethyl)-benzene; 428, 2,4,6-trimethylpyridine; 554, 2-(2-butoxyethoxy)-ethanol.

Analyzing the relative abundances of individual analytes, we observed some statistically significant differences in VOCs produced during the two life cycles but not between the two species. We performed a Mann-Whitney U test using Benjamini-Hochberg false discovery rate (FDR) correction comparing all spherule and mycelial VOCs and identified 43 that were significantly different in abundance (*P* < 0.05) (Fig. 2). Of the 43 volatiles, 32 of them had a higher abundance in spherules, compared to 11 in mycelia, which mirrors the overall difference in the sizes of the volatilomes (Fig. 1). Among the VOCs detected in higher abundances in one life cycle than in another were the aromatic hydrocarbon 1-methyl-3-(1-methylethyl)-benzene and the aldehyde hexanal, which are associated with mycelial cultures, while the alcohols 2-(2-butoxyethoxy)-ethanol and 2-hexen-1-ol, the ketone cyclohexanone, and the heteroaromatic 2,4,6-trimethylpyridine were associated with spherule cultures. There were no VOCs that were significantly different in relative abundance between *C. posadasii* and *C. immitis*.

**FIG 3:**
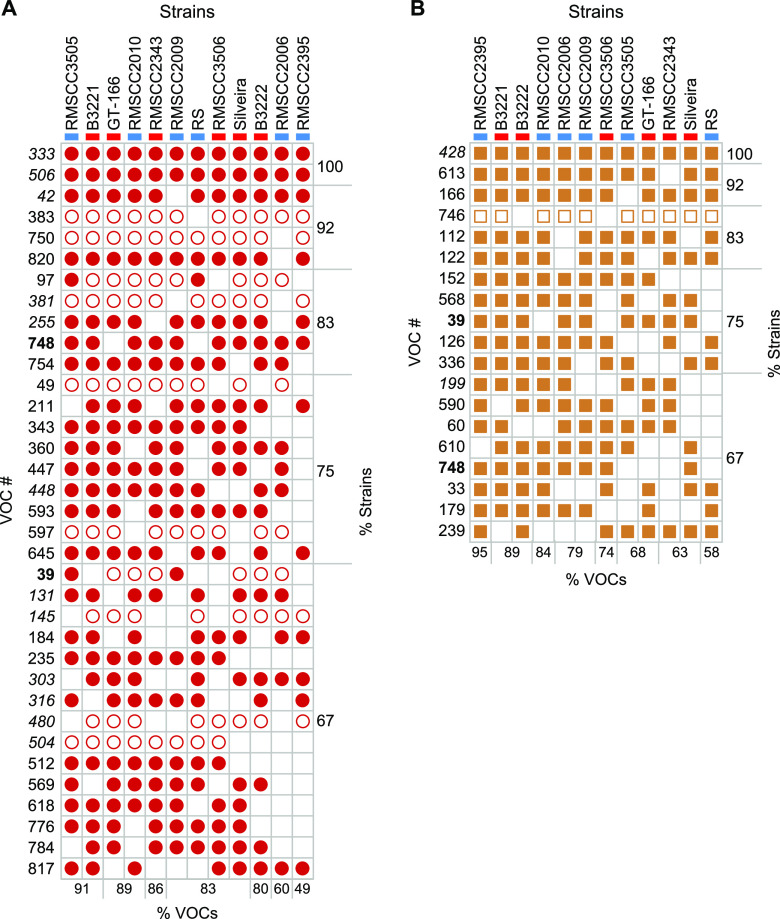
Volatiles detected in at least two-thirds of all *Coccidioides* strains in spherule (A) or mycelial (B) cultures, with VOCs in boldface type detected in both life cycles and those in italic type detected in statistically significantly different abundances between life cycles (*P* < 0.05). Open shapes indicate VOCs with at least a 2-fold-higher relative abundance than in medium blanks, and solid shapes indicate VOCs with at least a 10-fold-higher relative abundance. Strains are color-coded as blue for *C. immitis* and red for *C. posadasii*. The percentages of listed VOCs detected in each strain and the percentages of strains producing each VOC are noted at the bottom and right sides of each chart, respectively. Compound identifications: 97, acetic acid; 239, 1-hexanol; 255, 2-hexen-1-ol; 383, limonene; 428, 2,4,6,-trimethylpyridine; 820, 2-butyl-1-octanol.

Most (>70%) of the spherule and mycelial VOCs detected in this study were produced by four or fewer *Coccidioides* strains in each life cycle. Of the 272 spherule volatiles that were detected, only 35 (13%) were detected in at least two-thirds of all spherule cultures (Fig. 3A), 12 of which were also significantly more abundant in spherule than in mycelial cultures. Nineteen of 224 mycelial volatiles (8%) were detected in at least two-thirds of all mycelial samples (Fig. 3B), and only 2 were significantly different between life cycles (VOCs 199 and 428). However, both volatiles were produced in higher relative abundances in spherules. Only two VOCs, VOCs 39 and 748, were detected in two-thirds of both spherule and mycelial samples. Interestingly, the relative abundance of a VOC was usually consistent across all strains. For example, in every spherule culture in which limonene (VOC 383) was detected, it was present at a relative concentration that was between 2- and 10-fold higher than that in the medium (Fig. 3A).

### Multivariate analyses of the *Coccidioides* volatilome.

**FIG 4:**
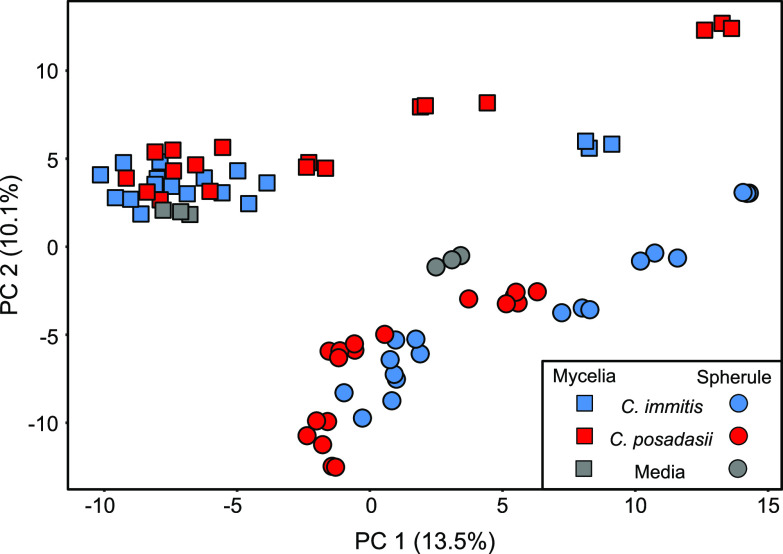
Principal-component analysis (PCA) score plot using 353 VOCs as features, produced by *C. immitis* (blue) and *C. posadasii* (red) when cultured under conditions that induced mycelial (squares) or spherule (circles) morphologies. Blank media are shown in gray. Fungal cultures and medium blanks were analyzed in triplicate, yielding 72 fungal and 6 blank observations. PCA score plots with the observations colored by strain are shown in Fig. S1 in the supplemental material.

Principal-component analysis (PCA), using the 78 cultures and medium blanks as observations and 353 *Coccidioides* VOCs as variables, shows that the volatilomes of individual strains and culture conditions are highly reproducible (Fig. S1A) and that they cluster by life cycle and not by species (Fig. 4). This observation is reinforced when we include only mycelia or spherules in the PCA, where no separation between species is observed (Fig. S1B and C), or include only *C. immitis* or *C. posadasii*, where we still observe a clear separation by life cycle (Fig. S1D and E). Evaluating these subsets of the data, some interesting variations between strains emerge. The type strain for *C. immitis* is RS; however, in both mycelial and spherule life cycles, we observe that its volatile metabolome is quite unique from those of the other *C. immitis* strains (Fig. S1). *C. posadasii* also has a metabolic outlier, RMSCC3506, but only during mycelial growth (Fig. S1B and E). These outliers cannot be fully explained by the size of the volatilomes as being abnormally large or small (Table S2).

10.1128/mSphere.00040-21.1 FIG S1 Principal-component analysis (PCA) score plot using VOCs as features, produced by 12 strains of *C. immitis* and *C. posadasii* when cultured under conditions that induced mycelial (square) or spherule (circle) morphologies. Fungal cultures and medium blanks were analyzed in triplicate. Fungal strains and the medium blanks are color-coded, as labeled in the key. (A) All samples as observations and 353 VOCs as features. (B) Mycelial cultures and blanks as observations and 224 VOCs as features. (C) Spherule cultures and blanks as observations and 272 VOCs as features. (D) *C. immitis* cultures and blanks as observations and 309 VOCs as features. (E) *C. posadasii* cultures and blanks as observations and 291 VOCs as features. Download FIG S1, JPG file, 0.5 MB. Copyright © 2021 Higgins Keppler et al. 2021 Higgins Keppler et al. https://creativecommons.org/licenses/by/4.0/ This content is distributed under the terms of the Creative Commons Attribution 4.0 International license.

**FIG 5:**
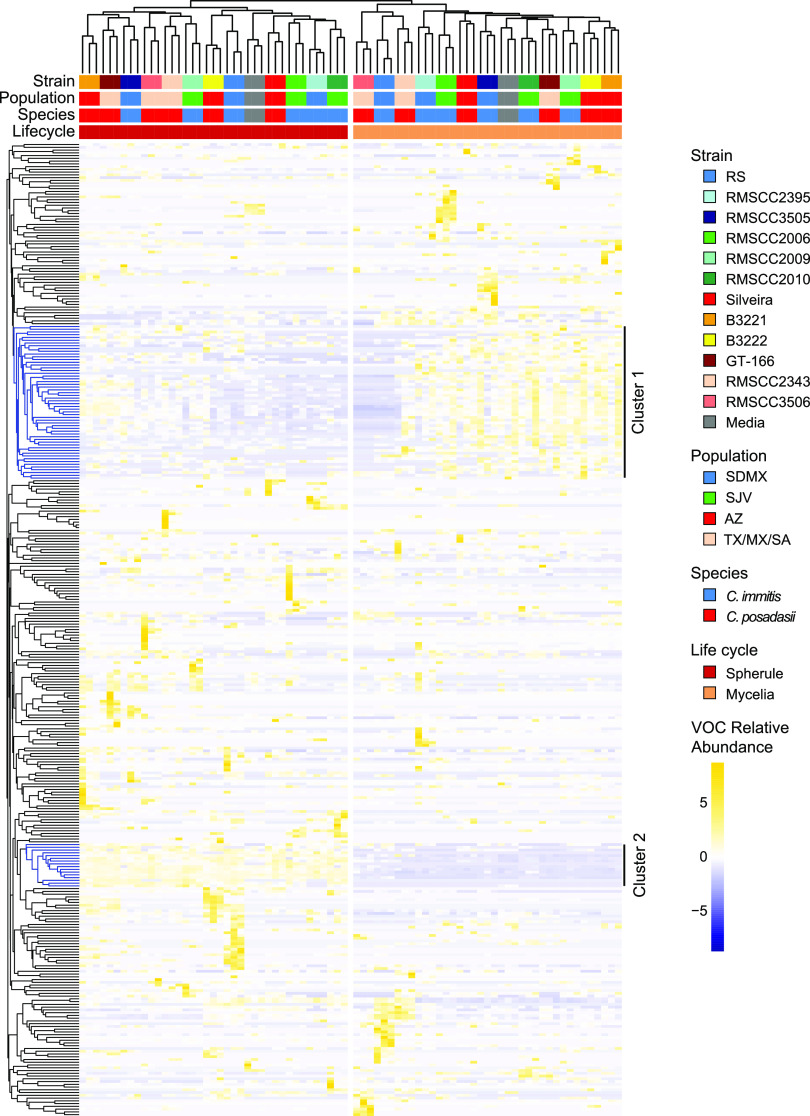
Hierarchical clustering analysis (HCA) of 72 fungal cultures and 6 medium blanks (columns) based on the relative abundances of 353 volatiles (rows). Clustering of fungal and blank samples was performed using Pearson correlations, and clustering of volatiles was performed using Euclidean distance, both with average linkages. Samples are color-coded by strain, population, species, and life cycle, as labeled in the key; medium blanks are in gray.

Although life cycle and strain differences in the *Coccidioides* volatilome are observable in the PCA, less than one-quarter of the total variance is captured by the first two principal components. Thus, we also used hierarchical clustering analysis (HCA) to compare the *Coccidioides* volatilomes (Fig. 5). The HCA reinforces our finding that the life cycle is the most significant contributor to the *Coccidioides* volatilome, and there are not global similarities in the volatilomes within each species or populations of the species. There are two main clusters of VOCs driving the separation of the mycelial and spherule life cycles in the HCA. Cluster 1 includes 56 VOCs, which are generally present in a higher abundance in mycelial samples than in spherule samples, 5 of which are significantly different between life cycles (*P* < 0.05 with Benjamini-Hochberg FDR correction) (Table S1). In cluster 1, seven compounds are identified: the hydrocarbon 3-undecene, the aromatic hydrocarbons 1-ethyl-4-methyl-benzene and 1-methyl-3-(1-methylethyl)-benzene, the alcohol 2-butyl-1-octanol, the aldehyde decanal, and the monoterpenes limonene and β-pinene. As in the PCA, we find that *C. immitis* RS and *C. posadasii* RMSCC3506 cluster separately when grown as mycelia, partially driven by the low abundances of VOCs from cluster 1. Cluster 2 includes 16 VOCs that are present in spherule samples while generally being absent or found at a lower abundance in mycelial samples, all of which are significantly different between life cycles (*P* < 0.05 with Benjamini-Hochberg FDR correction). Cyclohexanone (a ketone) and 2,4,6-trimethylpyridine (a heteroaromatic) are two VOCs in this cluster that we were able to identify.

## DISCUSSION

The genus *Coccidioides* is divided into two closely related and putatively allopatric species, with a strong phylogeographic signal and population substructure within each species (20). However, we find that the character of the *Coccidioides* volatilome is determined by life cycle rather than species. Within each species, the two life cycles have a relatively small overlap in their shared volatilome, with 27% and 31% being shared for *C. posadasii* and *C. immitis*, respectively (Fig. 1). While there were no volatiles that were significantly different in relative abundance between *C. posadasii* and *C. immitis*, we observed 43 statistically significant differences in the VOCs produced during the two life cycles (Fig. 2). This global difference between life cycles is the primary driver of separation that we observed in multivariate analyses using PCA (Fig. 4) and HCA (Fig. 5). These findings are in line with the large-scale transcriptional changes that occur when *Coccidioides* species transitions from mycelial to spherule growth: between 20% and 40% of the *Coccidioides* genes are differentially regulated during the shift between the environmental and parasitic life cycles (27–30). It has also been shown that the proteomes of the two life cycles are distinct, with statistically significant differences between mycelial and spherule life cycles (31). In contrast to the significant differences in the volatilomes between life cycles, we observed no uniform differences between the volatilomes of *C. posadasii* and *C. immitis*. There is a high degree of overlap (56 to 57%) between the species’ volatilomes when comparing the same life cycle (Fig. 1). This similarity is also visible in the PCA (Fig. 4; see also Fig. S1 in the supplemental material) and HCA (Fig. 5), as the volatilomes do not cluster by species even when we control for the life cycle. Global similarities between *C. posadasii* and *C. immitis* have also been demonstrated at the protein level (31, 32).

The lack of significant differences between the *C. posadasii* and *C. immitis* volatilomes is due, in part, to large differences observed between strains of the same species; the volatilome highlights some unique aspects of a few of the individual *Coccidioides* strains that we analyzed. For example, RS is the type strain of *C. immitis*; however, it appears to be a metabolic outlier during both life cycles (Fig. S1) but especially during mycelial growth (Fig. 5). Additionally, the mycelial life cycle of *C. posadasii* RMSCC3506 appears to be a metabolic outlier compared to the other strains grown as mycelia (Fig. 5; Fig. S1). These mycelial outliers appear to be driven, at least in part, by the lack of volatiles that make up cluster 1 in the HCA (Fig. 5). None of the 56 VOCs found in cluster 1, which are more abundant in mycelial than in spherule cultures, are detected in RMSCC3506 cultures, and only 2 are detected in RS mycelial cultures. These two strains also lack VOCs 39 and 748, which were detected in the majority of strains for both life cycles but absent from both life cycles of RS and mycelial cultures of RMSCC3506 (Fig. 3). Additionally, none of the 11 VOCs that were significantly more abundant in mycelial than in spherule cultures (Fig. 2 and Table S1) are detected in RMSCC3506, and only 2 are detected in RS. Beyond contributing to high levels of volatilome variance in *Coccidioides*, the significance of the strain-level differences relative to fungal biology or infection is not known. Variation in virulence among strains in murine models of coccidioidomycosis has been observed but has not been tested in the context of species or with a mechanistic hypothesis (33). In future studies of mouse models and human infections, we plan to investigate correlations between the *Coccidioides* volatilome and virulence and infection outcomes.

It should be noted that although the spherule and mycelial media had the same composition, we detected different VOCs in the headspace of the blanks (Fig. 4 and 5; Table S1). We interpret these differences as being the influence of the cultures on the blanks rather than the influence of the blank media on the cultures. Because the medium blanks were incubated and processed in parallel with their respective samples, we posit that the blank media absorbed volatiles from the culture environment (e.g., from fungal spherule or mycelial cultures in shared incubators), which introduced differences in their volatilomes. An analysis of fresh blank media (not incubated with the cultures) would be required to test this hypothesis. Our data indicate that the spherule media absorbed a higher abundance of compounds than the mycelial media (Table S1), which may be due to the spherule life cycle producing higher numbers and/or abundances of individual compounds. Therefore, it is possible that there are additional significant spherule volatiles that were not identified in this study since a VOC was defined as “detected” based on its relative abundance versus medium blanks. Future *in vitro* work needs to be mindful of volatile absorption by the cultures and blanks within a shared space such as an incubator.

The results from this study highlight two crucial factors that need to be considered in the development and translation of volatilomics (and other functional genomics data) into biomarkers. First, there can be a significant amount of intraspecies variation such that the volatilome of a single strain is not representative of the species as a whole (17, 34–36). Therefore, an analysis of multiple strains should be performed to identify a core volatilome, and the more genetically and phenotypically diverse the species, the more strains will need to be analyzed to determine the consensus set of volatiles produced by the majority of them. Second, our data show that the growth phase of the organism has a strong influence on the volatilome, and therefore, using *in vitro* culture conditions that can most closely replicate the *in vivo* environment and microbial growth phases may increase the likelihood of putative biomarker translation from bench to bedside. In addition to controlling the growth phase of dimorphic organisms, macronutrient, micronutrient, and oxygen availability should all be considered in the development of *in vitro* model systems that replicate the *in vivo* physiology of the pathogen (14, 17, 36–41). In this study, we observed 35 VOCs present in at least 67% of all spherule strains (Fig. 3); as this is the parasitic life cycle found within the host, we hypothesize that the most highly conserved spherule VOCs have the highest probability of translating to animal models and to human Valley fever breath biomarkers.

The first priority for diagnosing pneumonia etiology is determining whether the underlying cause is bacterial, fungal, or viral, as this will guide the selection of antibiotics, antifungals, or neither for treating the infection. We have initiated a study to directly compare coccidioidomycosis, bacterial pneumonia, and viral pneumonia in Arizona residents to determine the specificity of the *Coccidioides* spherule volatiles for diagnosing the fungal pneumonia that is endemic in this region of the United States. There have not yet been any human breath studies designed to compare multiple fungal and bacterial pneumonias to determine if there might also be a universal set of fungal breath volatiles, but given the limited overlap in the regions of the United States where fungal pneumonias are endemic (42), a set of universal fungal breath volatiles will not be required to substantially improve pneumonia diagnostics. It may be of clinical or epidemiological interest to differentiate coccidioidomycosis from other fungal pneumonias such as aspergillosis, blastomycosis, histoplasmosis, or cryptococcosis. Previous studies have shown that bacterial lung pathogens have unique and reproducible volatilomes that can be used to detect and identify organisms at the genus, species, or strain level (11), and therefore, differentiating fungal pneumonias is feasible.

### Conclusion.

The *Coccidioides* volatilome is strongly influenced by the saprobic versus parasitic life cycles, while the two species, *C. posadasii* and *C. immitis*, are indistinguishable by their volatile profiles, in part due to high strain-to-strain variability within the species. We identified 35 VOCs produced by the majority of the *Coccidioides* strains when grown as spherules, the parasitic form of the fungus found in Valley fever lung infections and in disseminated fungal disease. Our results show that as future efforts are made to develop biomarkers for *Coccidioides* and other mycoses caused by dimorphic fungi, it is vitally important to control for the fungal life cycle to represent parasitic growth in the host and to sample multiple strains of the pathogen to identify volatiles that are more highly conserved within the infectious genus or species.

## MATERIALS AND METHODS

### Fungal isolates and growth conditions.

The *Coccidioides* isolates used in the study were obtained from human patients and are listed in Table 1. All *Coccidioides* isolates were grown under biosafety level 3 (BSL-3) containment, under conditions that induce mycelial or spherule growth.

Fungal growth conditions were performed as described previously (18, 43) and are summarized here in brief. For mycelial growth, a 50-ml vented falcon tube containing 10 ml of RPMI medium (filter-sterilized RPMI 1640, 10% fetal bovine serum) was inoculated with a 1-cm by 1-cm 2× glucose yeast extract (GYE) agar plug for each strain. These plates were inoculated using 100 μl of a glycerol stock, spread across the plate, and cultured at 30°C for 2 weeks. Control RPMI medium was inoculated with a plug from sterile 2× GYE agar medium. Each sample, including the medium control, was prepared in triplicate. Cultures were grown on a shaking incubator at 150 rpm at 30°C for 96 h. For spherule cultures, a 50-ml vented falcon tube containing 10 ml of RPMI medium was inoculated to a final concentration of 1.0 × 10^5^ arthroconidia/ml in 1× phosphate-buffered saline (PBS). Arthroconidia were grown and harvested as previously described (18, 43). Strains RMSCC2343 and RMSCC3505 did not produce enough conidia to achieve 1.0 × 10^5^ arthroconidia/ml and were inoculated at 7.0 × 10^4^ and 4.0 × 10^4^ arthroconidia/ml, respectively. Control medium was inoculated with 1 ml of sterile 1× PBS. Cultures were grown on a shaking incubator at 150 rpm at 39°C in 10% CO_2_ for 96 h. Mycelial and spherule cultures were spun at 12,000 × *g* at 4°C for 10 min to pellet the cells. The supernatant was removed, placed in a Nanosep MF centrifugal device with a Bio-Inert membrane 0.2-μm spin filter, and centrifuged at 3,200 × *g* for 4 min. The filtrate was stored at −80°C until volatile metabolomics analysis.

To ensure the sterility of the filtrates for metabolomics analyses outside a BSL-3 containment facility, 10% of all sample filtrates were plated on 2× GYE and incubated at 30°C for 96 h to ensure the complete removal of viable pathogen particles. No growth was observed in any replicate.

### Volatile metabolomics analysis by SPME-GC×GC–TOFMS.

The *Coccidioides* species culture filtrates and medium blanks were allowed to thaw at 4°C overnight, and 2 ml was then transferred and sealed in sterilized 10-ml GC headspace vials with polytetrafluoroethylene (PTFE)-silicone septum screw caps. All samples were stored for up to 12 days at 4°C until analyzed. Samples were randomized for analysis. Volatile metabolite sampling was performed by solid-phase microextraction (SPME) using a Gerstel multipurpose sampler directed by Maestro software. Sample extraction and injection parameters are provided in Table S3 in the supplemental material (autosampler method). Volatile metabolite analysis was performed by two-dimensional gas chromatography–time of flight mass spectrometry (GC×GC-TOFMS) using Leco (St. Joseph, MI) Pegasus 4D and Agilent 7890 GC instruments. Chromatographic, mass spectrometric, and peak detection parameters are provided in Table S3 (GC×GC method and mass spectrometry method). An external alkane standard mixture (C_8_ to C_20_; Sigma-Aldrich, St. Louis, MO) was sampled multiple times for calculating retention indices (RIs). The injection, chromatographic, and mass spectrometric methods for analyzing the alkane standards were the same as the methods for the samples.

10.1128/mSphere.00040-21.5 TABLE S3 Parameters for HS-SPME and GC×GC-TOFMS analysis, and data processing and alignment. Download Table S3, PDF file, 0.2 MB. Copyright © 2021 Higgins Keppler et al. 2021 Higgins Keppler et al. https://creativecommons.org/licenses/by/4.0/ This content is distributed under the terms of the Creative Commons Attribution 4.0 International license.

### Processing and analysis of chromatographic data.

Data collection, processing, and alignment were performed using ChromaTOF software version 4.71 with the Statistical Compare package (Leco Corp.), using the parameters listed in Table S3 (data processing method).

Peaks were assigned a putative identification based on mass spectral similarity and RI data, and the confidence of those identifications is indicated by assigning levels 1 to 4 (with 1 being the highest) (44). Peaks with a level 1 identification were identified based on mass spectral and RI matches with external standards. Peaks with a level 2 identification were identified based on ≥800 mass spectral matches by a forward search of the NIST 2011 library and RIs that are consistent with the midpolar Rxi-624Sil stationary phase, as previously described (34), but using an RI range of 0 to 43% (empirically determined by comparing the Rxi-624Sil RIs for Grob mix standards to published polar and nonpolar values). Level 1, 2, and 3 compounds were assigned to chemical functional groups based upon characteristic mass spectral fragmentation patterns and second-dimension retention times, as previously described (35). Level 4 compounds have mass spectral matches of <600 or RIs that do not match previously published values and are reported as unknowns.

### Data postprocessing and statistical analyses.

The data postprocessing steps are depicted in Fig. S2. Before statistical analyses, compounds eluting prior to 358 s (acetone retention time) and siloxanes (i.e., chromatographic artifacts) were removed from the peak table. Peaks that were present in only one of the three biological replicates were imputed to zero for that sample, while missing values for peaks that were present in two out of three biological replicates were imputed to half of the minimum value across all biological replicates. The relative abundances of compounds across chromatograms were normalized using probabilistic quotient normalization (PQN) (45) in R version 3.4.3. The data were log_10_ transformed, intraclass correlation coefficients (ICCs) were calculated using R ICC package version 2.3.0, and peaks with an ICC of <0.75 were not further processed. Analytes were retained for further analysis if they were at least 2-fold more abundant in any *Coccidioides* culture than in the medium controls and were considered to have been detected in that culture. Principal-component analysis was performed using R factoextra package version 1.0.5 with the biological replicates as observations and the absolute peak intensities (mean centered and scaled to unit variance) as variables. The relatedness of samples based on their volatile metabolomes was assessed using hierarchical clustering analysis on Pearson’s correlation between isolates and the Euclidean distance between volatiles using R pheatmap package version 1.0.12. Geometric means of the biological replicates were calculated and used to determine the statistical difference in abundances between life cycles or species using a Mann-Whitney U test and Benjamini-Hochberg false discovery rate correction with an α value of 0.05, using R stats package version 3.5.3. The relative abundance of volatiles significantly different between life cycles was calculated by dividing the mean mycelial peak abundance by the mean spherule peak abundance.

10.1128/mSphere.00040-21.2 FIG S2 Data postprocessing workflow. Download FIG S2, JPG file, 0.3 MB. Copyright © 2021 Higgins Keppler et al. 2021 Higgins Keppler et al. https://creativecommons.org/licenses/by/4.0/ This content is distributed under the terms of the Creative Commons Attribution 4.0 International license.

### Data availability.

Metabolomic data (chemical feature peak areas and retention time information) included in this study are available at the NIH Common Fund’s National Metabolomics Data Repository (NMDR) website, the Metabolomics Workbench, at www.metabolomicsworkbench.org, where it has been assigned project identifier PR0001064 and study identifier ST001659 (https://doi.org/10.21228/M85H6W).
