# Supplementary material for: Life Cycle Dominates the Volatilome Character of Dimorphic Fungus Coccidioides spp
Source: mSphere. 2021 Apr 14;6(2):e00040-21. doi: 10.1128/mSphere.00040-21 (PMC8546678; doi:10.1128/mSphere.00040-21)
Supplement: TABLE S2 [file msphere.00040-21-st002.pdf]

Table S2.

Strain	Mycelia (n = 224)	Spherule (n = 272)
***C. posadasii* (n = 291)**		
Silveira*	51	61
B3221	56	122
B3222	55	80
RMSCC2343	69	80
RMSCC3506	51	77
GT-166	36	107
***C. immitis* (n = 309)**		
RS*	71	105
RMSCC2395	81	42
RMSCC3505	63	96
RMSCC2006	59	53
RMSCC2009	54	68
RMSCC2010	61	75
