# Supplementary figures and images for: Life Cycle Dominates the Volatilome Character of Dimorphic Fungus Coccidioides spp
Source: mSphere. 2021 Apr 14;6(2):e00040-21. doi: 10.1128/mSphere.00040-21 (PMC8546678; doi:10.1128/mSphere.00040-21)

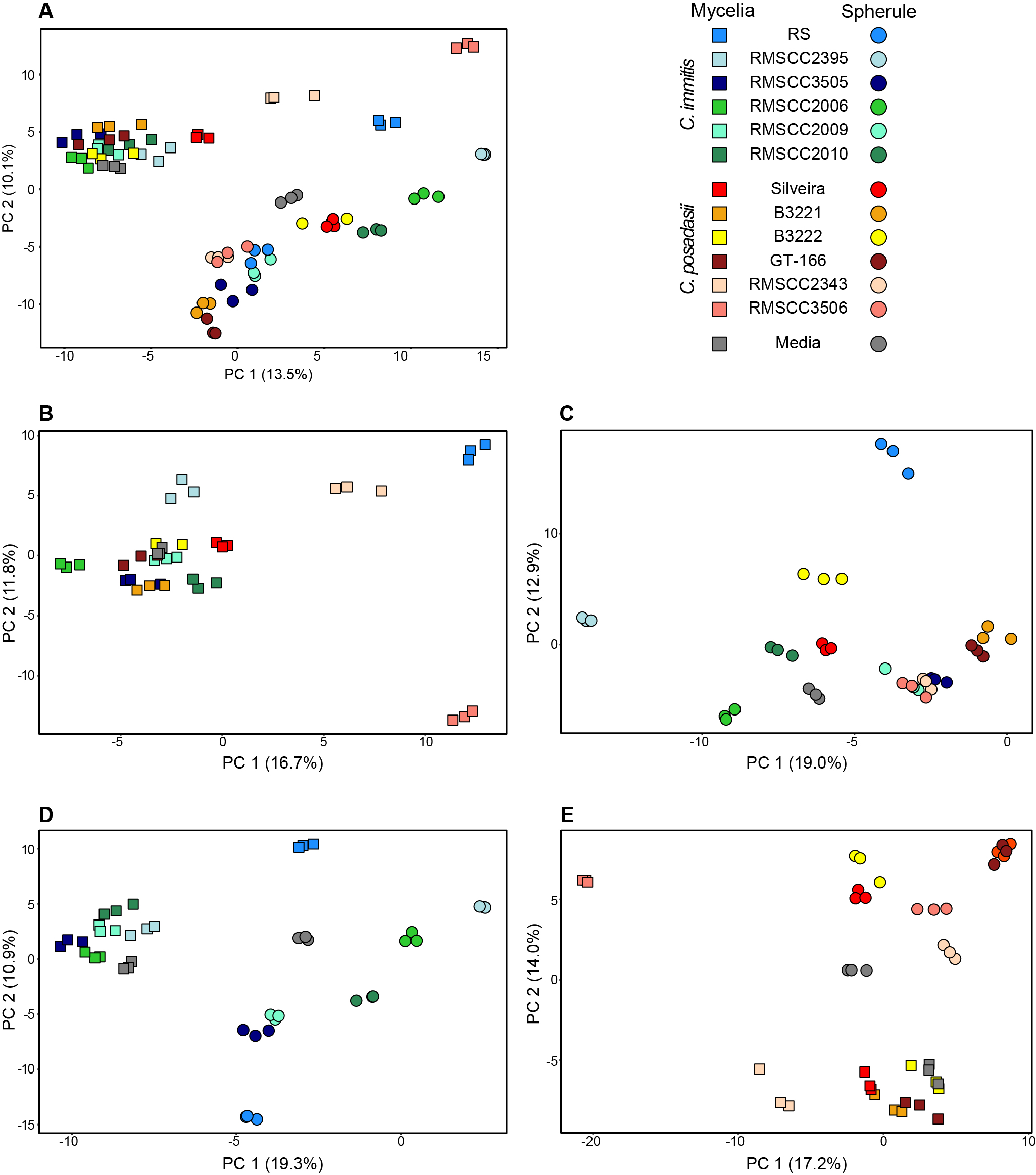

Supplement: FIG S1 [file msphere.00040-21-sf001.jpg]

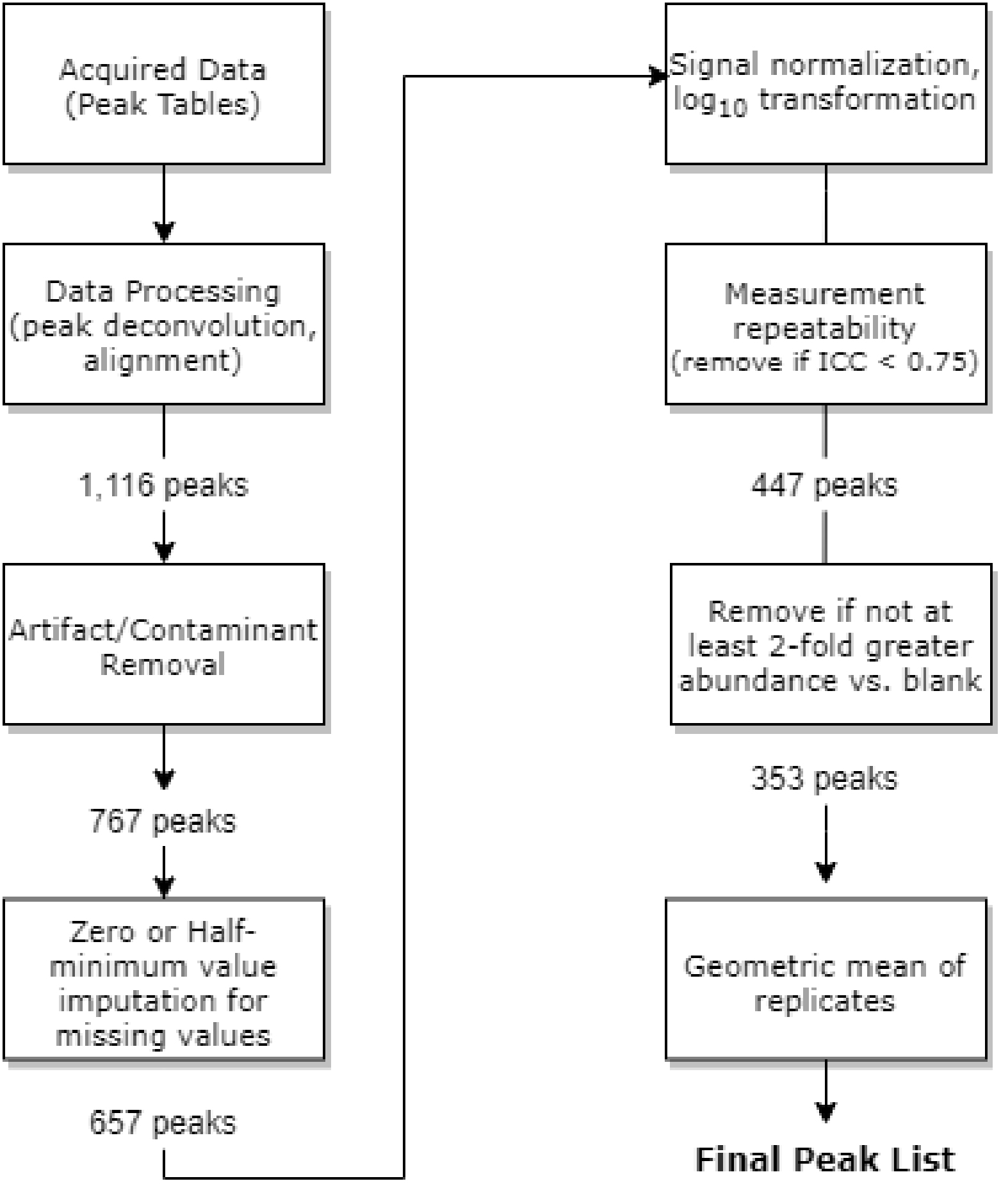

Supplement: FIG S2 [file msphere.00040-21-sf002.jpg]
