# Supplementary material for: Life Cycle Dominates the Volatilome Character of Dimorphic Fungus Coccidioides spp
Source: mSphere. 2021 Apr 14;6(2):e00040-21. doi: 10.1128/mSphere.00040-21 (PMC8546678; doi:10.1128/mSphere.00040-21)
Supplement: TABLE S3 [file msphere.00040-21-st003.pdf]

Table S3.

**AUTOSAMPLER METHOD**	
Instrument description	Gerstel® MPS Pro®
Software description	Gerstel® Maestro® (version 1.5.3.2)
**Sampling Parameters**	
Cooled tray temperature	4 °C
Solid phase microextraction (SPME)	Manufacturer: Supelco®<br>Fiber type: PDMS/CAR/DVB (1 cm; 50/30 µm)
Incubation time	5 min
Agitator parameters, incubation	Temperature: 50 °C<br>On time: 10 s<br>Off time: 1 s<br>Speed: 600 rpm
Agitation, sampling	On
Vial penetration	21 mm
Extraction time	10 min
Injection penetration	67 mm
Desorption time	180 s
**Inlet (CIS) Parameters**	
Initial temperature	250 °C
Equilibrium time	0.05 min
Initial time	0.10 min
Ramp rate	12 $°C \cdot s^{-1}$
End temperature	250 °C
Hold time	10.5 min
**GC×GC METHOD**	
Instrument description	Agilent® 7890B
Column configuration	Column 1: Rxi®-624Sil MS, 60 m × 0.25 mm × 1.4 μm<br>Column 2: Stabilwax®, 1 m × 0.25 mm × 0.5 μm
Carrier gas	Helium, 2 $mL \cdot min^{-1}$ (constant)
Front inlet type	Gerstel®
Front inlet mode	Splitless
Front inlet septum purge flow	1 $mL \cdot min^{-1}$
Front inlet septum purge time	300 s
Front inlet purge flow	50 $mL \cdot min^{-1}$
Front inlet total purge flow	52 $mL \cdot min^{-1}$
Oven equilibration time	5 s
Primary oven temperature ramp	Initial temperature: 35 °C<br>Initial time: 0.5 min<br>Ramp rate: 5 $C \cdot min^{-1}$

	Final temperature: 230 °C<br>Hold time: 5 min
Secondary oven temperature offset	+5 °C (relative to primary oven)
Modulator temperature offset	+15 °C (relative to secondary oven)
Modulation timing	Modulation period: 2.00 s<br>Hot pulse time: 0.50 s<br>Cold pulse time: 0.50 s
Transfer line temperature	250 °C
**MASS SPECTROMETRY METHOD**	
Instrument description	LECO® Pegasus® 4D
Use GC method total time for MS method total time	Yes
Acquisition delay	180 s
Filament active time	180 s to end of run
Start mass/End mass	35/400
Acquisition rate	100 spectra·s^{-1}
Optimized voltage offset	+50 V
Electron energy	-70 eV
Ion source temperature	250 °C
**DATA PROCESSING METHOD**	
Software description	LECO® ChromaTOF® and Statistical Compare (version 4.71.0.0)
Baseline tracking/Offset	Entire run/0.5 (through middle of noise)
Data points averaged for smoothing	Auto
First dimension peak width	12 slices
Mass spectral match required to combine	600
Second dimension peak width	0.15
Min. subpeak signal-to-noise (S/N) for	6
Integration approach	Traditional
Peak finding	S/N: 50<br>Number of apexing masses: 2
Mass spec libraries for searching	NIST 2011
Mass to use for area/height calculation	Unique mass
Alignment analyte match criteria	Spectral match mass threshold: 10<br>Minimum spectral similarity match: 600<br>Max. number of modulation periods apart: 3<br>Max. retention time difference (s): 0.2<br>S/N for second peak find: 5
Criteria for inclusion of analytes	Min. number of samples that contain analyte: 1
